# Factors associated with ABCDEF bundle implementation for critically ill patients: An international cross-sectional survey in 54 countries

**DOI:** 10.1177/20503121241312944

**Published:** 2025-01-09

**Authors:** Junpei Haruna, Takeshi Unoki, Keibun Liu, Kensuke Nakamura, Shigeaki Inoue, Osamu Nishida

**Affiliations:** 1 Department of Intensive Care Medicine, School of Medicine, Sapporo Medical University, Sapporo, Hokkaido, Japan; 2 Department of Acute and Critical Care Nursing, School of Nursing, Sapporo City University, Sapporo, Hokkaido, Japan; 3 Non-Profit Organization ICU Collaboration Network, Tokyo, Japan; 4 Critical Care Research Group, The Prince Charles Hospital, Brisbane, QLD, Australia; 5 Institute for Molecular Bioscience, The University of Queensland, Brisbane, QLD, Australia; 6 Department of Critical Care Medicine, Yokohama City University Hospital, Yokohama, Kanagawa, Japan; 7 Department of Emergency and Critical Care Medicine, Wakayama Medical University, Wakayama, Japan; 8 Emergency Medical Center, Wakayama Medical University Hospital, Wakayama, Wakayama Prefecture, Japan; 9 Department of Anesthesiology and Critical Care Medicine, Fujita Health University School of Medicine, Toyoake, Japan

**Keywords:** Intensive care unit liberation bundle, postintensive care syndrome, quality improvement, supportive care, ABCDEF bundle

## Abstract

**Objectives:**

This study investigated the implementation of the ABCDEF bundle and the factors associated with its implementation according to national income levels.

**Methods:**

This study is cross-sectional research. We conducted a secondary analysis of an international 1-day point-prevalence study that investigated the implementation of the ABCDEF bundle in critically ill patients. All patients admitted to the ICU were eligible. This study was conducted across 135 ICUs in 54 countries, including data from 664 patients. Outcomes were categorized according to the income level of the country (high-income, middle-income, and low-income countries) in which each ICU was located. A multilevel generalized linear model was developed to identify the factors associated with ABCDEF bundle implementation for each income level.

**Results:**

We identified 664 patients in 79 high-income countries, 278 in 26 middle-income countries, and 287 in 30 low-income countries ICUs. Implementation rates of the ABCDEF bundle were low for all income levels but varied. Few individuals completed the entire bundle on the survey date. Common factors associated with the implementation among all income levels were a multidisciplinary team approach for Element A (pain) and mechanical ventilation use for Element C (sedation), which were also associated with lower Element E (mobility). The existence of a protocol was frequently identified as a promoting factor associated with ABCDEF bundle implementation. The associated factors varied by income level; for example, dedicated intensivists were only identified in high-income countries, but not in middle-income countries or low-income countries.

**Conclusions:**

The overall low ABCDEF bundle implementation rates necessitate action. As factors associated with its implementation vary according to national income level, tailored strategies are essential for improving ICU care quality.

**Trial registration:**

NA.

## Introduction

Several ICU survivors have reported experiencing various symptoms after discharge, including impaired physical and cognitive functions and mental health-related disorders^1^; these symptoms are widely recognized as postintensive care syndromes (PICS).^2^ To prevent and treat PICS, the ABCDEF bundle is recommended as evidence-based guidance for clinicians to coordinate multidisciplinary patient care in the ICU.^3^

The ABCDEF bundle includes the following elements: assessment, prevention, and management of pain; spontaneous awakening (SAT) and spontaneous breathing trials (SBT); choice of analgesia and sedation; delirium; early mobility and exercise; and family engagement and empowerment. The ABCDEF bundle was designed to improve the quality of ICU care with the aim of liberating ICU patients from harmful ICU-related impacts, including pain, agitation, oversedation, delirium, longer mechanical ventilation, and immobility.^4^ A study employing the ABCDEF bundle as an intervention observed notable improvements in posttraumatic stress disorder and health-related quality of life in the intervention group after 1 year.^5^ Furthermore, good adherence to the ABCDEF bundle has been reported to correlate with positive patient outcomes, such as a reduction in the incidence of ventilator-associated pneumonia.^6^ Consequently, enhancing the ABCDE bundle implementation rate is important when treating critically ill patients.

Recent studies have described several general methods for implementing the ABCDEF bundles.^3^ However, an important factor; the national income levels, has not been taken into account. The optimal functioning of an ICU depends on a delicate balance of material resources, organizational structure, staff availability, and training.^7^ Increasing pressure on healthcare costs worldwide, particularly during the COVID-19 pandemic, has significantly limited the availability of healthcare resources, exerting varying degrees of impact on individuals with different income levels.^8^ Given that financial and structural situations vary greatly between low-income countries (LICs) and high-income countries (HICs), strategies to improve the quality of ICU care associated with the ABCDEF bundle may also differ by income level.^9^ Therefore, it is imperative to develop strategies tailored to each country’s income level rather than to seek a general “one-size-fits-all” strategy. However, to the best of our knowledge, no study has examined the factors associated with a high level of ABCDEF bundle implementation according to income level.

Thus, this study aimed to investigate the implementation of the ABCDEF bundle and identify the factors associated with its implementation, according to national income level.

## Methods

### Design and setting

This study was conducted in accordance with the principles of the Helsinki Declaration of 1975. This study was approved by the Ethics Committee of Saiseikai Utsunomiya Hospital (IRB Protocol Number: 2020-69, approved 12 December 2020). We conducted a secondary analysis of an international 1-day point-prevalence study that investigated the ABCDEF bundle implementation in critically ill patients, on 27 January 2021.^10^ The need for written informed consent was waived by the IRB of Saiseikai Utsunomiya Hospital. The study was conducted in accordance with the STROBE guidelines.

From 8–26 January 2021, the study committee collaborated with regional/country coordinators to send invitation letters to the Indian Society of Critical Care Medicine, Korean Society of Critical Care Medicine, and other local and regional/national networks to recruit ICUs (S1 Appendix). All ICUs that complied with the study guidelines were enrolled, and no exclusion criteria were applied. Each participating ICU registered one representative, hospital name, and country name to confirm the authenticity of the data (S1 Appendix). The invitation letter, created using Google Forms (Google Inc., Mountain View, CA, USA), included a brief introduction to the study, ethical considerations, and links to registration. It also contained the URL of a web page created in Google Forms (Google Inc.) that described the study in detail (https://form.jsea2005.org/isiic-II-study/). The study committee requested representatives from all registered ICUs to provide hospital and ICU background data upon receiving the invitation letter. These data were collected using Google Forms, from 20 January to 26 January 2021, before the commencement of the study, and served as a presurvey for this research (S2 Appendix). After completing the presurvey, representatives from all registered ICUs automatically received facility registration numbers. On 27 January 2021, an evidence-based ICU care survey was sent to all ICU representatives. All ICU representatives were asked to enter their preissued facility registration numbers as response to the first question. Only participants who entered this information were able to complete the survey (see S3 Appendix). The purpose of the facility registration number was to link hospital and ICU background data with the evidence-based ICU care survey data since all data were collected in an anonymous manner. The study’s co-authors (KL, KN, SI, and ON) reviewed the survey questions in advance, and the co-investigator physicians and nurses listed in the acknowledgments further assessed them for internal validity. The survey URL was accessible between 27 January and 30 January 2021.

### Participants

The World Bank categorizes countries into four income groups: low, lower-middle, upper-middle, and high-income. For the purpose of this study, countries were divided into three categories according to their income levels: HICs, MICs, and LICs. This study considered World Bank-defined low- and lower-middle countries as LICs and upper-middle income countries as MICs. This classification was applied to the responding ICUs as of 1 October 2023, using data from the World Bank’s country and lending groups (source: https://datahelpdesk.worldbank.org/knowledgebase/articles/906519-world-bank-country-and-lending-groups).

### Variables

Hospital and ICU background data included protocols for each ABCDEF bundle element; the party primarily responsible for ABCDEF bundle implementation; total number of hospital beds; total number of ICU beds; number of ICU beds allocated for COVID-19; nurse-to-patient ratio; dedicated ICU professionals; hospital type; ICU-responder identity; and tele-ICU availability.

The evidence-based ICU care survey included data on pain and sedation in ICU patients, the presence or absence of ICU care modality and mobilization goals, and the implementation status of each ABCDEF bundle element. Detailed operational information on these elements is provided in S4 Appendix.

Patient attribute recorded included the presence of COVID-19 infections, the ICU length of stay on the survey date, sex, age, body mass index (BMI), usage of medical devices, neuromuscular blockers, vasoactive drugs, analgesics, continuous sedatives, and prone positioning for specified durations.

### Outcomes

The primary outcome was the implementation rate of the individual elements and of the entire ABCDEF bundle. Secondary outcomes were factors associated with the implementation of each element in the ABCDEF bundle.

### Statistical analysis

The background, operational structure, and policies of the participating hospitals and ICUs, as well as the demographics of the ICU patients on the survey date, were categorized, according to the national income level of the country in which the ICU was located, into three income levels: HICs, MICs, and LICs. First, descriptive analysis was conducted. Continuous variables were presented as medians and interquartile ranges (IQRs). Categorical variables are presented as numbers and percentages. We used the Kruskal–Wallis test for evaluating continuous variables and Fisher’s exact test for evaluating categorical variables. No missing data existed in this study.

The outcomes were also demonstrated by income levels. Factors associated with bundle implementation were identified based on national income level. Multivariate analysis was conducted using a multilevel generalized linear model (GLM) with binomial distribution and an identity link function. A multilevel GLM was implemented, based on the country’s income levels. Explanatory variables influencing ABCDEF bundle implementation were identified from previous studies^4,11^; these variables included mechanical ventilation use, COVID-19, ICU length of stay, nurse-to-patient ratio, the presence of an intensivist and physical therapists dedicated to the ICU, use of a multidisciplinary team approach with primary responsibility for implementation of the ABCDEF bundle, and the presence of a specific protocol. In the analysis, variables for which the number of patients assigned to a category was too small (⩽20) to generate an appropriate model were excluded from the GLM. The results of the multilevel GLM are presented as odds ratios (OR) and 95% confidence intervals (95% CIs). Because this was a secondary analysis, the sample size was not calculated. Statistical significance was set at *p* < 0.05. All analyses were performed using R (The R Foundation for Statistical Computing, Vienna, Austria) and STATA software (Stata Corp, College Station, TX, USA).

## Results

### Overview of hospitals and ICUs

**Figure 1.**
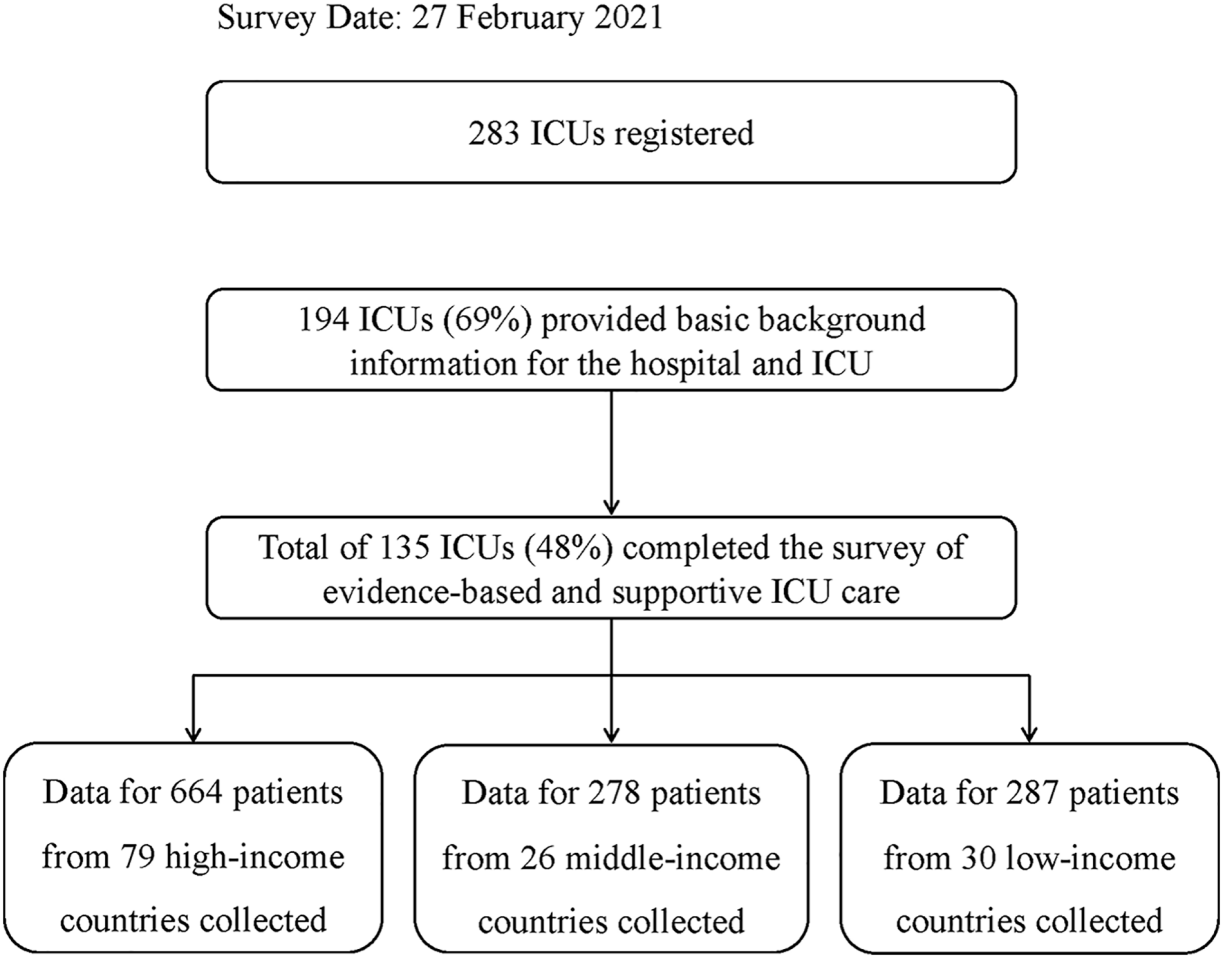
Study flow chart.

The survey was completed in 135 ICUs across 54 countries. Based on predefined income levels, 79 ICUs were located in HICs, 26 in MICs, and 30 in LICs. Information was registered for 664, 278, and 287 patients, respectively (Figure 1).

The background, operational structure, and policies of the participating hospitals and ICUs varied by national income level (Table 1), number of hospital beds, and types of hospitals and ICUs. LICs had a higher number of ICU beds (median: 23.5, IQR: 10.8–31.3) compared to MICs (median: 11, IQR: 10–20) and HICs (median: 14, IQR: 9–21). LICs also allocated more ICU beds for COVID-19 patients (median: 8.5, IQR: 4–18.5, *p* = 0.025). Regarding staffing, LICs had a higher nurse-to-patient ratio, with 69.2% of their ICUs maintaining a 1:1 ratio, compared to 19.0% in HICs. Additionally, LICs (50.0%) were more likely to employ respiratory therapists than MICs (19.2%) and HICs (20.3%), whereas HICs had a greater proportion of dedicated pharmacists (59.5%). Tele-ICU services were also more commonly available in LIC ICUs (13.3%, *p* = 0.024).

**Table 1.** Comparison of background, operational structure and policies of participating hospitals and ICUs by income levels.

Variable	Over all (*n* = 135)	High-income countries (*n* = 79)	Middle-income countries (*n* = 26)	Low-income countries (*n* = 30)	*p*-Value
Total number of hospital beds (beds), *n* (%)					
x < 200	21 (15.6)	4 (5.1)	10 (38.5)	7 (23.3)	<0.001
200 ⩽ x < 400	19 (14.1)	6 (7.6)	5 (19.2)	8 (26.7)	
400 ⩽ x < 600	27 (20.0)	19 (24.1)	1 (3.8)	7 (23.3)	
600 ⩽ x < 800	26 (19.3)	21 (26.6)	0 (0.0)	5 (16.7)	
x ⩾ 800	42 (31.1)	29 (36.7)	10 (38.5)	3 (10.0)	
Type of hospital, *n* (%)					
University hospital	56 (41.5)	35 (44.3)	7 (26.9)	14 (46.7)	0.257
University affiliated hospital	35 (25.9)	15 (19.0)	11 (42.3)	9 (30.0)	
Community hospital	35 (25.9)	23 (29.1)	7 (26.9)	5 (16.7)	
Others	9 (6.7)	61 (7.6)	1 (3.8)	2 (6.7)	
Type of ICU, *n* (%)					
Medical-surgical mixed ICU	103 (76.3)	68 (86.1)	16 (61.5)	19 (63.3)	<0.001
Medical ICU	14 (10.4)	2 (2.5)	5 (19.2)	7 (23.3)	
Surgical ICU including cardiac surgery	13 (9.6)	7 (8.9)	3 (11.5)	3 (10.0)	
Pediatric ICU	2 (1.5)	0 (0.0)	1 (3.8)	1 (3.3)	
Other	3 (2.2)	2 (2.5)	1 (3.8)	0 (0.0)	
Total number of ICU beds (beds), median (IQR)	14 [10–25]	14 [9–21]	11 [10–20]	23.5 [10.8–31.3]	0.042
Number of ICU beds allocated for COVID-19 (beds), median (IQR)	4 [2–10]	4 [1–8]	4.5 [0–11.3]	8.5 [4–18.5]	0.025
Nurse-to-patient ratio, *n* (%)					
1	42 (31.1)	15 (19.0)	18 (69.2)	9 (30.0)	
2	72 (53.3)	55 (69.6)	3 (11.5)	14 (46.7)	
3	13 (9.6)	6 (7.6)	2 (7.7)	5 (16.7)	
>4	8 (5.9)	3 (3.8)	3 (11.5)	2 (6.7)	
Dedicated ICU professionals, *n* (%)					
Intensivist	123 (91.1)	74 (93.7)	23 (88.5)	26 (86.7)	0.450
Physiotherapist	72 (53.3)	43 (54.4)	17 (65.4)	12 (40.0)	0.157
Occupational therapist	15 (11.1)	8 (10.1)	1 (3.8)	6 (20.0)	0.145
Respiratory therapist	36 (26.7)	16 (20.3)	5 (19.2)	15 (50.0)	0.005
Pharmacist	67 (49.6)	47 (59.5)	9 (34.6)	11 (36.7)	0.024
Tele-ICU availability, *n* (%)	6 (4.4)	1 (1.3)	1 (3.8)	4 (13.3)	0.024
Presence of protocols for each ABCDEF bundle element, *n* (%)					
Element A: pain management	69 (51.1)	38 (48.1)	11 (42.3)	20 (66.7)	0.135
Element B: spontaneous awakening trial (SAT) management	47 (34.8)	29 (36.7)	4 (15.4)	14 (46.7)	0.043
Element B: spontaneous breathing trial (SBT) management	64 (47.4)	39 (49.4)	9 (34.6)	16 (53.3)	0.325
Element C: sedation management	66 (48.9)	43 (54.4)	8 (30.8)	15 (50.0)	<0.001
Element D: delirium management	54 (40.0)	36 (45.6)	8 (30.8)	10 (33.3)	0.287
Element E: early mobility and exercise	61 (45.2)	43 (54.4)	8 (30.8)	10 (33.3)	0.037
Element F: family engagement and empowerment	13 (9.6)	10 (12.7)	0 (0.0)	3 (10.0)	0.165
No protocol associated with the ABCDEF bundle	24 (17.8)	17 (21.5)	0 (0.0)	7 (23.3)	0.030
Primary responsibility for the ABCDEF bundle implementation					
None	25 (18.5)	16 (20.3)	6 (23.1)	3 (10.0)	0.642
Multidisciplinary team approach	39 (28.9)	22 (27.8)	7 (26.9)	10 (33.3)	
Intensivist	53 (39.3)	31 (39.2)	8 (30.8)	14 (46.7)	
Nurse	9 (6.7)	6 (7.6)	2 (7.7)	1 (3.3)	
Physiotherapist	8 (5.9)	4 (5.1)	1 (3.8)	0 (0.0)	
Respiratory therapist	1 (0.7)	0 (0.0)	1 (3.8)	0 (0.0)	

Data in table are presented as median [Interquartile range] or number (%). The *p*-value is based on comparisons between the three income groups (HICs, MICs, LICs) by Kruskal–Wallis test. COVID-19, Corona Virus Infectious Disease, emerged in 2019; ICU, intensive care unit; IQR, interquartile range.

Protocols for Elements A and B were used more frequently in LICs, whereas protocols for Elements D and E were used in HICs. The use of the Element C protocol was lowest among MICs across all income levels. The Element F protocol was rarely used, in ICUs in any income level. Specific protocols for all elements of the ABCDEF bundle were least available in MICs; however, at least one protocol was present in the MICs. Approximately 20% of HICs and LICs did not have any protocols for the ABCDEF bundle. A multidisciplinary team and an intensivist dedicated to the ICU were the most frequently assigned the primary responsibility for implementing the ABCDEF bundle in the ICU, regardless of the national income level.

### Patient demographics

Table 2 displays patient demographics according to national income levels. HICs included fewer patients with COVID-19 infection and shorter ICU length of stay (3 days [IQR 3–13 days]), whereas LICs showed the largest proportion of patients with COVID-19 and the longest ICU stay (7 days [IQR 4–25 days]). The majority of patients in ICUs in countries of all income levels were male. The distribution of age and BMI varied across national income levels, with relatively older patients observed in HICs and younger patients in LICs. Around 70% of patients in each group required either invasive or noninvasive mechanical ventilation. Extracorporeal membrane oxygenation (6.0%) and renal replacement therapy (10.7%) were more commonly provided in HICs.

**Table 2.** Comparison of demographics of patients by income levels.

Variable	Overall (*n* = 1229)	High-income countries (*n* = 664)	Middle-income countries (*n* = 278)	Low-income countries (*n* = 287)	*p*-Value
Presence of COVID-19 infection, *n* (%)	602 (49.0)	274 (41.3)	159 (57.2)	169 (58.9)	<0.001
ICU length of stay (days), median [IQR]	7 [3–14]	3 [3–13]	6 [3–10]	7 [4–25]	0.005
Sex (male), *n* (%)	816 (66.4)	452 (68.1)	178 (64.0)	186 (64.8)	0.395
Age (years), *n* (%)					
x < 20	58 (4.7)	46 (6.9)	5 (1.8)	7 (2.4)	<0.001
20 ⩽ x < 50	239 (19.4)	84 (12.7)	79 (28.4)	76 (26.5)	
50 ⩽ x < 60	222 (18.1)	107 (16.1)	63 (22.7)	52 (18.1)	
60 ⩽ x < 70	313 (25.5)	151 (22.7)	78 (28.1)	84 (29.3)	
70 ⩽ x < 80	282 (22.9)	190 (28.6)	38 (13.7)	54 (18.8)	
x ⩾ 80	115 (9.4)	86 (13.0)	15 (5.4)	14 (4.9)	
Body mass index (kg/m^2^), *n* (%)					
x < 18.5	94 (7.6)	72 (10.8)	4 (1.4)	18 (6.3)	<0.001
18.5 ⩽ x < 25	460 (37.4)	285 (42.9)	84 (30.2)	91 (31.7)	
25 ⩽ x < 30	373(30.3)	196 (29.5)	92 (33.1)	85 (29.6)	
30 ⩽ x < 35	194 (15.8)	71 (10.7)	66 (23.7)	57 (19.9)	
x ⩾ 35	108 (8.8)	40 (6.0)	32 (11.5)	36 (12.5)	
Usage of medical devices, *n* (%)					
Invasive mechanical ventilation	701 (57.0)	398 (59.9)	159 (57.2)	144 (50.2)	0.02
Noninvasive mechanical ventilation	187 (15.2)	78 (11.7)	51 (18.3)	58 (20.2)	<0.001
Extracorporeal membrane oxygenation	48 (3.9)	40 (6.0)	2 (0.7)	6 (2.1)	<0.001
Renal replacement therapy	122 (9.9)	71 (10.7)	29 (10.4)	22 (7.7)	0.34
Patients receiving continuous use of neuromuscular blockers, *n* (%)	178 (14.5)	94 (14.2)	47 (16.9)	37 (12.9)	0.375
Patients receiving continuous use of vasoactive drugs, *n* (%)	394 (32.1)	240 (36.1)	82 (29.5)	72 (25.1)	0.002
Patients receiving continuous use of analgesics, *n* (%)	649 (52.8)	380 (57.2)	142 (51.1)	127 (44.3)	<0.001
Patients receiving continuous use of sedatives, *n* (%)	589 (47.9)	336 (50.6)	130 (46.8)	123 (42.9)	0.082
Patients receiving prone positioning, *n* (%)	226 (18.4)	100 (15.1)	65 (23.4)	61 (21.3)	0.004
Presence of a target or goal applied to ICU patients on the survey date, *n* (%)					
Pain	456 (37.1)	277 (41.7)	68 (24.5)	111 (38.7)	<0.001
Sedation	673 (54.8)	391 (58.9)	139 (50.0)	143 (49.8)	0.007
Mobilization	557 (45.3)	321 (48.3)	121 (43.5)	115 (40.1)	0.05

ICU: intensive care unit; IQR: interquartile range.

Data in table are presented as median [Interquartile range] or number (%). The *p*-value is based on comparisons between the three income groups (HICs, MICs, LICs) by Kruskal–Wallis test. COVID-19, Corona Virus Infectious Disease, emerged in 2019.

Continuous use of vasoactive drugs, analgesic agents, and sedative agents was more frequent in HICs (36.1%, 57.2%, and 50.6%, respectively), whereas prone positioning was performed more frequently in MICs (23.4%) and LICs (21.3%). The targets for pain, sedation, and mobilization were achieved more frequently in HICs than they were in MICs or LICs.

### Primary outcome—implementation of evidence-based ICU care

A comparison of the implementation rates of each ABCDEF factor by country of income for the primary outcomes is presented in Table 3. HICs had significantly higher rates for Elements A, C, and D, compared to other income groups (*p* < 0.001) (Element A: 66.6% vs 49.3% vs 53.0%, Element C: 61.9% vs 54.7% vs 30.3%, and Element D: 49.5% vs 19.8% vs 23.7%. Elements B and E were implemented at a rate of <20% for all income levels. Among patients undergoing invasive mechanical ventilation, less than 10% of patients in ICUs of all national income levels received Element E. The protocol related to element F was very limited in all countries (Table 1), whereas the implementation rate was significantly more frequent in MICs (49.3%) than in LICs (24.4%) and HICs (10.8%), of which many cases of Element F were conducted via only in MICs. The stringent visitation restriction was frequently applied in ICUs in HICs (66.7%).

**Table 3.** Comparison of implementation of the ABCDEF bundle.

Variable	Overall (*n* = 1229)	High-income countries (*n* = 664)	Middle-income countries (*n* = 278)	Low-income countries (*n* = 287)	*p*-Value
Primary outcome: implementation of each element in the ABCDEF bundle in each income group					
Element A, *n* (%)	731 (59.4)	442 (66.6)	137 (49.3)	152 (53.0)	<0.001
Element B: both SAT and SBT^a^	67 (12.4)	39 (12.9)	8 (6.7)	20 (17.2)	0.045
SAT under continuous sedation, *n* (%)^b^	98 (16.6)	60 (17.9)	12 (9.2)	26 (21.1)	0.026
SBT during mechanical ventilation, *n* (%)^c^	130 (10.6)	77 (11.6)	18 (6.5)	35 (12.2)	0.012
Element C, *n* (%)	650 (52.9)	411 (61.9)	152 (54.7)	87 (30.3)	<0.001
Element D, *n* (%)	452 (36.8)	329 (49.5)	55 (19.8)	68 (23.7)	<0.001
Element E, *n* (%)	175 (14.2)	93 (14.0)	32 (11.5)	50 (17.4)	0.128
Element E during mechanical ventilation, *n* (%)^c^	44/701 (15.9)	24/398 (6.0)	6/159 (3.8)	14/144 (9.7)	0.098
Element F, *n* (%)	279 (22.7)	72 (10.8)	137 (49.3)	70 (24.4)	<0.001
Element F which was conducted via online, *n* (%)	150 (12.2)	28 (4.2)	95 (34.2)	27 (9.4)	<0.001
Visiting arrangements for family to meet patients in the ICU in each income group					
Meeting not allowed, *n* (%)	630 (51.3)	443 (66.7)	123 (44.2)	64 (22.3)	<0.001
In person, *n* (%)	307 (25.0)	145 (21.8)	81 (29.1)	81 (28.2)	0.022
Visiting through the glass outside the room, *n* (%)	36 (2.9)	4 (0.6)	19 (6.8)	13 (4.5)	<0.001
Using electronic device (using a monitor such as phone/video), *n* (%)	269 (21.9)	76 (11.4)	59 (21.2)	134 (46.7)	<0.001
Implementation of complete or adjusted ABCDEF bundle by income countries					
Performing an entire of the ABCDEF bundle, *n* (%)^a^	2 (0.4)	0	0	2 (1.7)	0.026
Performing any combinations of five of six elements: A, B, C, D, E, and F, *n* (%)^a^	15 (2.8)	6 (2.0)	0 (0.0)	9 (7.8)	<0.001
Performing an entire of the ABCDEF bundle except B, *n* (%)^d^	25 (2.0)	11 (1.7)	5 (1.8)	9 (3.1)	0.317
Performing any combinations of four of five elements: A, C, D, E, and F, *n* (%)^d^	76 (6.2)	35 (5.3)	18 (6.5)	23 (8.0)	0.266

ICU: intensive care unit; SAT: spontaneous awakening trials; SBT: spontaneous breathing trials.

Data in table are presented as number (%). The *p*-value is based on comparisons between the three income groups (HICs, MICs, LICs) by Kruskal–Wallis test.

a COVID-19, coronavirus disease 2019. The targeted ICU patients are those who receive continuous sedation and mechanical ventilation at the same time. A total number of those patients are 539, including 303 patients from HICs, 116 patients from LICs, and 120 from MICs. Percentages were calculated by dividing by these numbers of sedated and ventilated patients.

b The targeted ICU patients are those who receive continuous sedation. A total number of those patients are 589, including 336 patients from HIC, 123 patients from LIC, 130 patients and from MIC. Percentages were calculated by dividing by these numbers of sedated patients.

c The targeted ICU patients are those who receive mechanical ventilation. A total number of those patients are 701, including 398 patients from HIC, 144 patients from LIC, and 159 patients from MIC. Percentages were calculated by dividing by these numbers of ventilated patients.

d The targeted ICU patients are all ICU patients on the survey date.

The stringent visitation restrictions were frequently applied in ICUs in HICs (66.7%). Online methods were frequently employed in LICs. Implementation of the entire ABCDEF bundle was rarely achieved in ICUs associated with any national income levels.

Factors associated with the implementation of each element of the ABCDEF bundle by national income level.

The factors associated with the implementation of the ABCDEF bundle for each income level are presented in Tables 4–10. The associated factors varied across income levels, with the multidisciplinary team approach and the existence of a specific protocol frequently identified as significant. Additionally, the presence of an intensivist dedicated to the ICU was a key factor in HICs.

**Table 4.** Independent factors associated with the implementation of element A by income.

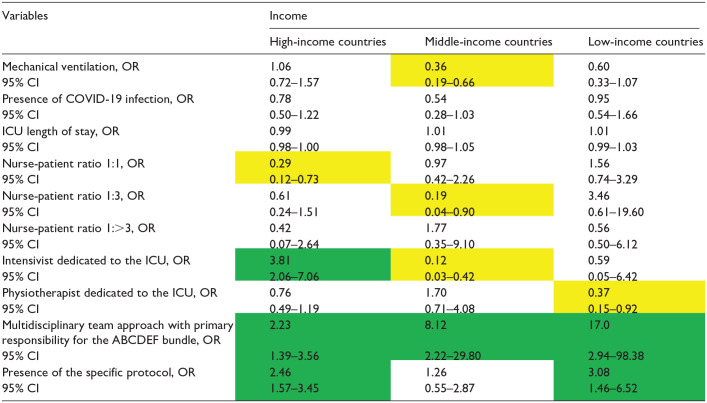

COVID-19: coronavirus disease 2019; ICU: intensive care unit.

Data in table are presented as odds ratio (OR), 95% confidence interval (CI). Green indicates a significantly higher OR; yellow indicates a significantly lower OR, and [–] denotes non-estimation.

**Table 5.** Independent factors associated with the implementation of element B SAT by income.

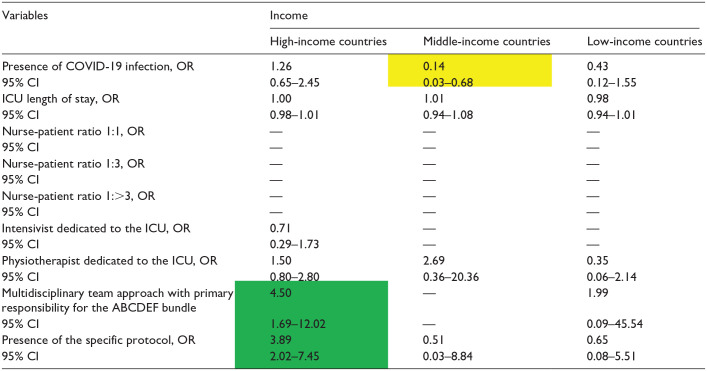

COVID-19: coronavirus disease 2019; ICU: intensive care unit.

Data in table are presented as odds ratio (OR), 95% confidence interval (CI). Green indicates a significantly higher OR; yellow indicates a significantly lower OR, and [—] denotes nonestimation.

**Table 6.** Independent factors associated with the implementation of element B SBT by income.

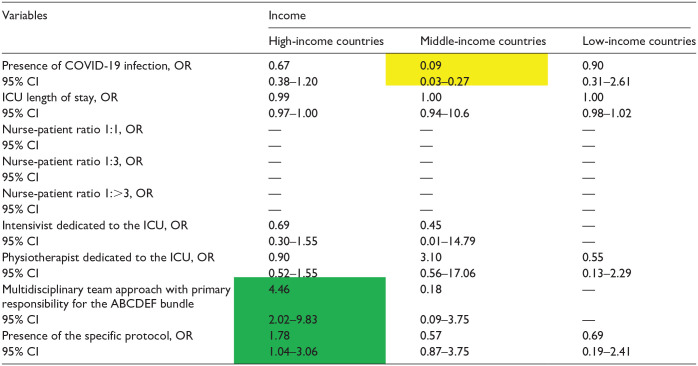

COVID-19: coronavirus disease 2019; ICU: intensive care unit.

Data in table are presented as odds ratio (OR), 95% confidence interval (CI). Green indicates a significantly higher OR; yellow indicates a significantly lower OR, and [—] denotes nonestimation.

**Table 7.** Independent factors associated with the implementation of the element C by income.

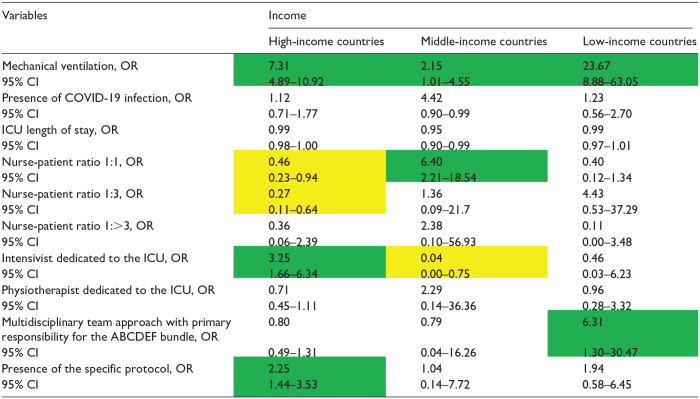

COVID-19, coronavirus disease 2019; ICU, intensive care unit.

Data in table are presented as odds ratio (OR), 95% confidence interval (CI). Green indicates a significantly higher OR; yellow indicates a significantly lower OR, and [–] denotes non-estimation.

**Table 8.** Independent factors associated with the implementation of the element D by income.

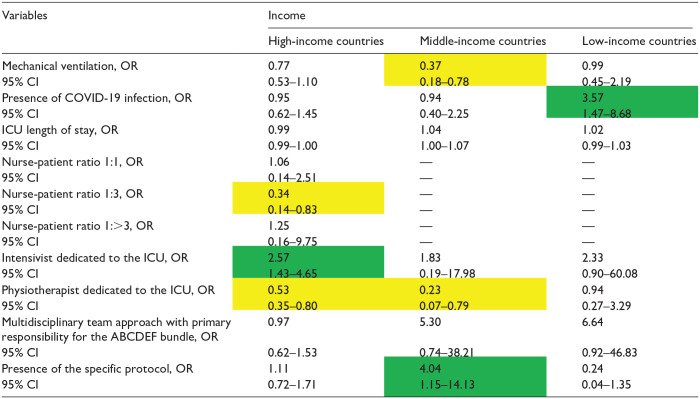

COVID-19: coronavirus disease 2019; ICU: intensive care unit.

Data in table are presented as odds ratio (OR), 95% confidence interval (CI). Green indicates a significantly higher OR; yellow indicates a significantly lower OR, and [—] denotes nonestimation.

**Table 9.** Independent factors associated with the implementation of the element E by income.

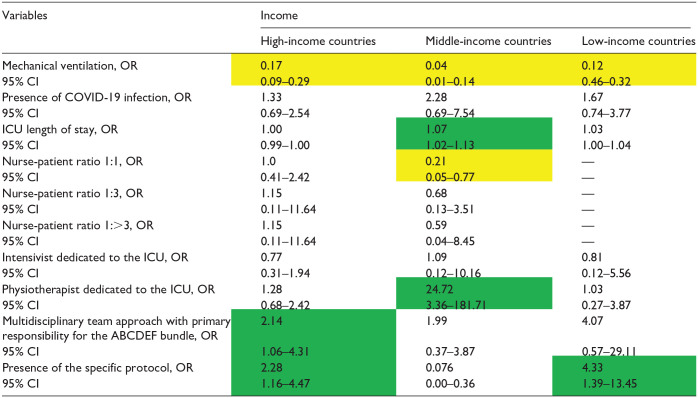

COVID-19: coronavirus disease 2019; ICU: intensive care unit.

Data in table are presented as odds ratio (OR), 95% confidence interval (CI). Green indicates a significantly higher OR; yellow indicates a significantly lower OR, and [—] denotes non-estimation.

**Table 10.** Independent factors associated with the implementation of the element F by income.

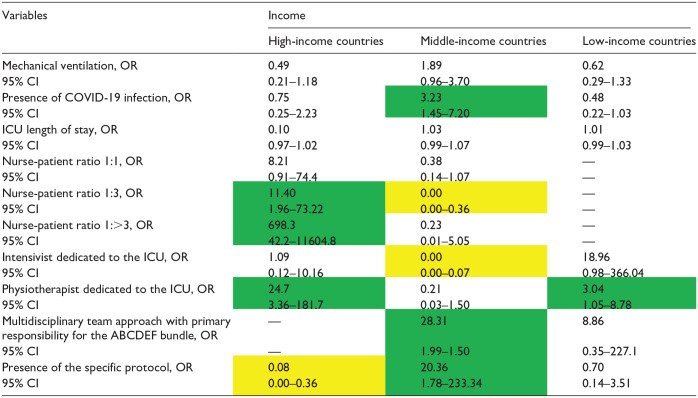

COVID-19: coronavirus disease 2019; ICU: intensive care unit.

Data in table are presented as odds ratio (OR), 95% confidence interval (CI). Green indicates a significantly higher OR; yellow indicates a significantly lower OR, and [—] denotes nonestimation.

### Element A

For Element A (Table 4), the multidisciplinary team approach was associated with better bundle implementation, regardless of the national income level (HICs, OR: 2·23, 95% CI: 1.39–3.56; MICs, OR: 8.12 95% CI: 2.22–29.80; LICs, OR: 17, 95% CI: 2.94–98.38). The existence of a specific protocol was also an associated factor in HICs and LICs, with ORs of 2·46 (95% CI: 1.57–3.45) and 3·08 (95% CI: 1.46–6.52), respectively. The presence of an intensivist dedicated to the ICU was associated only in HICs, with an OR of 3.81 (95% CI: 2.06–7.06).

### Element B (SAT)

For Element B (Table 5), the multidisciplinary team approach and the presence of a specific protocol were associated with higher bundle implementation in HICs (OR: 3·08 95% CI: 1.46–6.52; OR: 3.89, 95% CI: 2.02–7.45, respectively). No other factors were associated with implementation of Element B.

### Element B (SBT)

The multidisciplinary team approach and the presence of the specific protocol were also only associated with higher implementation of Element B of the bundle in HICs (OR: 4.46, 95% CI: 2.02–9.83; OR: 1.78, 95% CI: 1.04–3.06, respectively; Table 6).

### Element C

Mechanical ventilation was associated with higher implementation of Element C, irrespective of national income levels (OR: 7.31, 95% CI: 4.89–10.92; OR: 2.15, 95% CI: 1.04–4.55; OR: 23.67, 95% CI: 8.88–63.05) (Table 7). A multidisciplinary team approach and the existence of an intensivist dedicated to the ICU were independent factors for HICs, whereas the presence of a specific protocol was an independent factor for LICs in terms of implementation of Element C (Table 7).

### Element D

The existence of a specific protocol was an independent factor in MICs, whereas a multidisciplinary team approach was not associated with the implementation of Element D at any income level (Table 8). The presence of an intensivist dedicated to the ICU was significantly associated with the implementation of Element D in HICs (Table 8).

### Element E

Patients requiring mechanical ventilation had poor implementation of Element E across all income levels. (HIC OR: 0.17, 95% CI: 0.09–0.29; MIC OR: 0.04, 95% CI: 0.01–0.14; LIC OR: 0.12, 95% CI: 0.46–0.32; respectively) (Table 9). In HICs, a multidisciplinary team approach was associated with better bundle implementation (OR: 2.14; 95% CI: 1.06–4.31), but not in MICs and LICs. In both HICs and LICs, the existence of a specific protocol was an independent factor for implementation of Element E (OR: 2.28, 95% CI: 1.16–4.47; OR: 4.33, 95% CI: 1.39–13.45; respectively). In MICs, the presence of a physical therapist was associated with higher implementation of Element E of the bundle.

### Element F

The multidisciplinary team approach (OR: 28.31, 95% CI: 1.99–1.50) and the presence of the specific protocol (OR: 20.36, 95% CI: 1.78–233.34) were only associated with higher implementation of Element F in MICs (Table 10). The presence of a physical therapist was associated with higher implementation of Element F in HICs and LICs (Table 10).

## Discussion

This study revealed that the implementation rates of the individual elements of the ABCDEF bundle, and of the entire ABCDEF bundle, varied but were low overall, irrespective of national income levels. Among all income levels, few patients experienced implementation of the entire ABCDEDF bundle by the survey date, strongly indicating the need to improve the quality of ICU care to enhance patient outcomes. This study also highlighted the variability in factors associated with the implementation of the ABCDEF bundle according to income level. Indicating the importance of using tailored approaches or strategies to facilitate evidence-based care according to income level.

In this study, the implementation rates of the ABCDEF bundle were lower, regardless of national income level, than that noted in a survey conducted before the COVID-19 pandemic.^12^ The implementation of the ABCDEF bundle not only improves mortality and reduces ICU stay duration^4^ but is also considered to be effective in preventing PICS.^3^ The implementation rates of Elements A, C, and D of the bundle varied significantly according to the national income level, with better implementation observed in HICs. Various factors, including differences in the healthcare systems associated with different national income levels and patient characteristics, might be associated with these results.^13^ Therefore, it is crucial to identify the barriers to implementing the bundle and to investigate practically modifiable or addressable factors to facilitate the implementation rates of the ABCDEF bundle according to the relevant indicator such as income level.^14^

One of the barriers to implementing the bundle was the use of mechanical ventilation. Mechanical ventilation was associated with Element C and E implementation across all income levels. Although the implementation rate of Element C differed significantly in HICs/MICs/LICs, mechanical ventilation was identified as an independent factor at all income levels. The implementation rate of Element C in LICs was the lowest. This may be due to the paucity of experience and education regarding mechanical ventilation in LICs. Although it has long been pointed out that mechanical ventilators are a barrier to the implementation of Element E,^11^ this study demonstrated that this fact applies to ICUs across nations of all income levels. To improve bundle adoption rates, as shown in Table 9, approaches should be tailored to each income level, such as using a multidisciplinary team approach, using specific protocols for HIC, a dedicated ICU physiotherapist for MIC, and establishing specific protocols for LIC.

Several studies have highlighted that using an evidence-based multidisciplinary team approach for ABCDEF bundle implementation provides favorable patient outcomes. For example, coordinated efforts by nurses and respiratory therapists in SAT and SBT are reportedly safe and effective in facilitating ventilator weaning.^15^ In addition, a dedicated ICU physiotherapist and nurse reviewed the criteria for initiating patient mobilization and collaborated during the mobilization task to increase the rate of early mobilization.^16^ Therefore, dedicating a multidisciplinary team to specific roles is important for successful bundle implementation.^17^ Based on the above, ICU healthcare professionals can more effectively implement the ABCDEF bundle by combining their skills and expertise, potentially leading to improved patient outcomes. However, it is important to be aware of the time and expense involved in training and employing a multidisciplinary team approach.^18^

The presence of specific protocols was found to be related to improvements in elements A, SAT, SBT, C, and E in HICs, elements D and E in MICs, and elements A and E in LICs. To standardize and implement the ABCDEF bundle, protocols for SAT/SBT,^19^ delirium,^20^ and early mobilization^16^ are very useful, irrespective of the income level, because of their easy availability and low cost. Although numerous barriers to introduce a protocol have been reported,^11^ the barriers may vary according to the hospital (e.g., size, policy, structure, available resources, staffing). Therefore, the hospital-specific barriers should be examined before introducing a protocol.^21^

A previous study reported that having intensivists dedicated to the ICU is associated with improved clinical outcomes.^22^ The intensivist’s leadership is needed to ensure safe and consistent bundle deployment through team communication and daily care plan development.^23^ The presence of intensivists also helps to ensure proper bundle implementation and appropriate evidence-based patient care.^24^ This study showed that the presence of intensivists, only in HICs, may have been associated with higher rates of implementation of the ABCDEF bundle. Thus, HICs differ from MICs/LICs in terms of intensivist education levels and specialty systems. Nearly all HICs organize their intensive care education systems based on funding capacities and abundant resources.^9^ In contrast, LICs and MICs often experience a critical shortage of physicians with specialized training in intensive care, due to the paucity of national funding, which is the most important barrier to intensive care development.^25^ Moreover, numerous ICUs lack a critical care specialist and consequently lack efficient protocols and a multidisciplinary team approach, which are essential components of a high-quality ICU.^26^ MICs and LICs require programs for educating intensivists, which would facilitate implementation of ABCDEF bundles to improve ICU care quality, as seen in HICs.

Maximizing quality intensive care requires focusing on each country’s economic and healthcare status, including human resources, ICU operational cost management, and ICU resource use within the healthcare system. For example, physicians’ expertise, nurse-to-patient ratios, and the presence of other medical professionals in ICU vary widely between LICs/MICs and HICs.^8^ In HIC ICUs, the nurse-to-patient ratio is generally in the range of 1:1–1:2.^27,28^ In LICs, the nurse-to-patient ratio can be as high as 1:25, reflecting a critical shortage of nursing staff.^29^ This stark disparity underscores the profound challenges faced by LICs in delivering effective and safe ICU care, particularly compared to HICs where staffing levels are more robust. Thus, economic factors can significantly affect ICU capacity. The factors affecting the ABCDEF bundle implementation differed considerably between LICs and HICs in our study. Therefore, an income-specific approach may be necessary to facilitate ABCDEF bundle implementation, depending on the resources and structures of each ICU or hospital.

### Strengths and limitations

To the best of our knowledge, no previous study has investigated the implementation rates and factors associated with the implementation of the ABCDEF bundle by income level globally. However, some limitations exist. First, a selection bias may have been significantly influenced by the limited number of participating countries. We minimized this effect by adjusting for it through multivariate analysis using several covariates for each element of the ABCDEF bundle. Second, our findings may have been influenced by the COVID-19 pandemic. Although the survey date captured the peak of the COVID-19 wave in Japan,^30^ the results must be interpreted in light of the fact that the impact of the COVID-19 pandemic differed from country to country and depended on the timing of the survey. Although the study was conducted after peak periods in many regions, the lingering effects on staffing, resource availability, and burnout may have influenced the findings. The pandemic’s impact on healthcare workers persisted beyond its peak, and this may have contributed to variations in ABCDEF Bundle adoption, particularly in resource-limited settings. Third, a point-prevalence study cannot establish a causal relationship; it merely reflects the current situation at the participating sites. Therefore, a prospective international study that collects daily ABCDEF bundle data would be required. Fourth, we did not investigate potential confounding factors related to the implementation of the ABCDEF bundle, such as the backgrounds of healthcare professionals in each country. In addition, we were unable to collect data on underlying diseases, illness severity, or precise individual patient data. Fifth, as this was a secondary analysis, the sample size was not calculated in advance. Consequently, there may be limitations on the statistical significance and generalizability of the results obtained. However, we used multivariate analysis as a statistical method and made efforts to minimize bias as much as possible. This is a measure to ensure the reliability of the results.

### Implications for clinical practice

We discovered that strategies for implementing the ABCDEF bundle at high rates varied according to national income levels. Despite potential imbalances in the distribution of healthcare resources due to each country’s social and economic conditions, a multidisciplinary team approach and establishment of a specific protocol can be applied to all income levels to facilitate implementation of the ABCDEF bundle. However, a tailored approach that considers the differences in national income levels, potentially implicating the differences in healthcare resources and ICU capacity, is also essential for growing the culture of implementing the ABCDEF bundle across all income levels.

## Conclusion

This study found that implementation of the ABCDEF bundle was low overall, but varied according to national income level. In order to improve the implementation rate of the ABCDEF bundle, it is important to develop tailored strategies that take into account the challenges and resource limitations specific to each income level.

## Supplemental Material

sj-docx-1-smo-10.1177_20503121241312944 – Supplemental material for Factors associated with ABCDEF bundle implementation for critically ill patients: An international cross-sectional survey in 54 countries Supplemental material, sj-docx-1-smo-10.1177_20503121241312944 for Factors associated with ABCDEF bundle implementation for critically ill patients: An international cross-sectional survey in 54 countries by Junpei Haruna, Takeshi Unoki, Keibun Liu, Kensuke Nakamura, Shigeaki Inoue and Osamu Nishida in SAGE Open Medicine
