# Supplementary material for: Factors associated with ABCDEF bundle implementation for critically ill patients: An international cross-sectional survey in 54 countries
Source: SAGE Open Med. 2025 Jan 9;13:20503121241312944. doi: 10.1177/20503121241312944 (PMC11713948; doi:10.1177/20503121241312944)
Supplement: sj-docx-1-smo-10.1177_20503121241312944 – Supplemental material for Factors associated with ABCDEF bundle implementation for critically ill patients: An international cross-sectional survey in 54 countries [file sj-docx-1-smo-10.1177_20503121241312944.docx]

**e - Appendix 1: List of Collaborators and Site Investigators**

National societies / networks which endorsed this study

Indian Society of Critical Care Medicine (ISCCM): President of the Indian Society of Critical Care Medicine, Dr. Dhruva Chaudhry; Korean Society of Critical Care Medicine (KSCCM): President of the Korean Society of Critical Care Medicine, Dr. Sang Hyun Kwak; ICU Recovery Network; Japanese Society of Early Mobilization (JSEM); Japanese Society of Education for Physicians and Trainees in Intensive Care (JSEPTIC); Infectious Diseases Association for Teaching and Education in Nippon (IDATEN); Emergency Medicine Alliance (EMA);

Social Networking Service: Facebook, Twitter

Web-site (https://forms.gle/sUJfLDpYoJ9nHDZr7)

National coordinators outside of Japan

The role of national coordinators is to recruit hospitals in their local area through their own personal networks or local societies to which they belong.

| Region | Country | Name | Institution |
| --- | --- | --- | --- |
| Europe | Germany | Peter Nydahl, RN | Nursing Research, Department of Anesthesiology and Intensive Care Medicine, University Hospital of Schleswig-Holstein, Kiel, Germany. |
| Africa | Libya | Muhammed Elhadi, MBBCh | Faculty of Medicine, University of Tripoli, Tripoli, Libya |
| Asia | India | Mohan Gurjar, M.D. | Department of Critical Care Medicine, Sanjay Gandhi Post Graduate Institute of Medical Sciences (SGPGIMS), India |
| Asia | Malaysia | Be Kim Leong, M.D., MRehabMed | Department of Rehabilitation Medicine, Sarawak General Hospital, Kuching, Sarawak |
| Asia | Korea | Chi Ryang Chung, M.D., Ph.D. | Department of Critical Care Medicine, Samsung Medical Center, Sungkyunkwan University School of Medicine, Seoul, Korea |
| Asia | Singapore | Balachandran Jayachandran, M.Phty | Rehabilitation Department, Woodlands Health Campus, Singapore |

ISIICⅡ committee information

Office address: Level 2, 1-2-12, Kudankita, Chiyoda-ku, Tokyo, 102-0073

Office email: [isiic2@jsea2005.org](about:blank)

Office TEL: +81-3-3556-5585

Staff: Tomoya Kuroda

ISIIC Ⅱ Steering committee members

Keibun Liu, Kensuke Nakamura, Hajime Katsukawa, Muhammed Elhadi, Peter Nydahl, Eugene Wesley Ely, Sapna R. Kudchadkar, Shigeaki Inoue, Osamu Nishida

Online questionnaire creation

Data security, revision and management:

Tomohiro Sonoo: Hitachi General Hospital, Hitachi, Ibaraki, TXP Medical Co. Ltd., Tokyo), Takumi Ochiai: TXP Medical Co. Ltd., Tokyo

Site investigators

One representative from each ICU was registered. If there are names of several representatives in one hospital, it means that there are different ICUs registered, with different functions and locations or the principal representative needs other investigators’ help because of the overwhelming situation during the COVID-19 pandemic.

| Country | Hospital | Representatives |
| --- | --- | --- |
| ALBANIA | University Hospital Center " Mother Theresa ", Tirana | Ilir Ohri  Dariel Thereska |
| ALGERIA | Ibn Sina Hospital | Kouidri Khadidja |
| ANDORRA | Hospital Nostra Senyora de Meritxell | Antoni Margarit Ribas |
| ARGENTINA | Hospital Italiano de Buenos Aires  Centro Gallego de Buenos Aires  Luis Lagomaggiore | Nicolas A. Gemelli  Luis Alejandro Boccalatte  Graciela Zakalik |
| AUSTRALIA | The Royal Children's Hospital Melbourne | Shinya Miura |
| BANGLADESH | Ibn Sina Medical College Hospital  United Hospital  Asgar Ali Hospital, Dhaka | Tarikul Hamid  Mohammed Salah Uddin  Md Motiul Islam |
| BRAZIL | Hospital municipal ruth cardoso  Hospital Memorial Arthur Ramos  Hospital Carvalho Beltrão  Complexo Hospitalar Regional Deputado Janduhy Carneiro | Pedro Salomão  Igor Lima Buarque  Gustavo Mendonça Ataíde Gomes  Rebeca Dias Rodrigues Araújo |
| COLOMBIA | Hospital San Vicente Fundación Medellín | Edward Blandón |
| CYPRUS | Near East University Hospital | Heyam Almezghwi |
| EGYPT | Assiut University Hospital  Prince Sultan Hospital  Minia University Hospitals  Sohag general hospital  Aswan University Hospital  Berkit el sabaa general hospital  Alexandria Main University Hospital  Kafr Elsheikh University hospital  Tanta University Hospital  El Safa Hospital  Ain-Shams university  National Cancer Institute, Cairo University  Berkit el sabaa general hospital  Elmabara hospital  Benha University Hospital | Aliae AR Mohamed Hussein  Aliaa Abd Rabo  Nehal Gamal Omar  Ahmed Mohammed Abu-Elfatth  Ibrahim Fawzy ELgouhary Abdelfattah  Yasmin K. NasrEldin  Monica Dobs  Islam Galal  Mohamed Hellmy Zaki Zagho  Hanan M. Hemead  Sarah M. Hemead  Eslam M. Khalaf  Mostafa Mahmoud Tayeb  Mohammad Elbahnasawy  Muhammad Hamad  Ahmed Mahmoud Mohamed Saad  Wafaa Abdelsalam  Mohamed Elbahnasawy  Ahmed Y Azzam  Ahmed K. Awad  Galal Ghaly  Mohamed Hellmy Zaki Darwish Zagho  Neama mashhout  Ahmed Elshafey  Ahmed Abdelmoein  Ahmed Abdelsadek |
| EL SALVADOR | General Hospital. Salvadoran Social Security Institute. | Carlos E. Orellana-Jimenez |
| FRANCE | Hôpital Nord Franche-Comté  Groupe Hospitalier Sud Ile-de-France, Hôpital de Melun-Sénart | Fernando Daniel Berdaguer Ferrari  Sebastien Jochmans |
| GREECE | Athens General Hospital of Evaggelismos  Saint Paul ("Agios Pavlos") General Hospital  University Hospital of Ioannina | Irini Patsaki  Theodoros Aslanidis  Georgios Papathanakos |
| GUATEMALA | Hospital Roosevelt | Zonia Guzman |
| INDIA | Sir Gangaram Hospital  Tagore Hospitel JALANDHR  Sanjay Gandhi Postgraduate Institute of Medical Sciences (SGPGIMS)  Virinchi Hospital  Apollo hospitals  Royalcare superspeciality hospital  Health City Hospital  BKL Walawalkar Hospital, Chiplun  VPMH Hospital, Pune  Santosh Medical College Hospital .Ghajiabad  Believers Church Medical College Hospital  Manipal Hospital  Yashoda Hospital, Somajiguda, Hyderabad,  AIIMS, Patna  AIIMS Rishikesh | Niraj Tyagi  dr ziyokov joshi  Mohan Gurjar  Srinivas Samavedam  Saroj Kumar Pattnaik  Lakshmikanthcharan  Chandana Sarma  Amol Hartalkar  Sheetal Hartalkar  Anil Kumar  Sanjo Sunny  Gautham M Raju  Kaladhar S  Divendu Bhushan  Nidhi Gupta |
| IRAN | Imam Reza Hospital | Ata Mahmoodpoor |
| IRAQ | Al-Hilla Teaching Hospital  Arzheen Private Hospital  Babylon Maternal and Children  Zafaraniyah General Hospital  Alhakim General Hospital in Najaf | Ali Al-Isawi  Hayder Yousif Alhasan  Haiderbareh  Rand Hussein  Maytham Al-Juaifari |
| ITALY | Sant'Andrea Hospital  Ospedale "Magalini" - Villafranca di Verona | Monica Rocco  Plinio Calligaro |
| JORDAN | Irbid Speciality Hospital  Jordanian Royal Medical Services- King Hussein Medical Center  Islamic Hospital | Almu'atasim Khamees  Amro Mohammad Abuleil    Bourhan Alrayes |
| JAPAN | Teine Keijinkai hospital  Kobe University Hospital  Kagoshima City Hospital  Nagasaki University Hospital  Sapporo City General Hospital  Tokushima University Hospital  Ageo Central General Hospital  Tokushima Prefectural Central Hospital  Shinshu University Hospital  Okayama Saiseikai General Hospital  Niigata University Hospital  Fukuyama City Hospital  Okinawa Kyodo Hospital  Japanese Red Cross Kyoto Daiichi Hospital  Urasoe General Hospital  Mie University Hospital  Hyogo Prefectural Amagasaki General Medical Center  Saga University Hospital  Okayama University Hospital  Fukuoka University Hospital  Chugoku Rosai Hospital  Shinkomonji Hospital  International University of Health and Welfare Narita Hospital  Wakayama Medical University  St. Marianna University, School of Medicine, Yokohama-city Seibu Hospital  University of Tokyo Hospital  Minaminagano Medical Center Shinonoi General Hospital  Hirosaki University Hospital  Saiseikai Utsunomiya Hospital  Osaka General Medical Center  National Hospital Organization Tokyo Medical Center  Yokohama Municipal Citizen's Hospital  National Hospital Organization Disaster Medical Center  Obihiro-Kosei Hospital  Nishijima Hospital  Hamamatsu University Hospital  Otsu City Hospital  Hitachi General Hospital  Tokyo Medical and Dental University  Japanese Red Cross Maebashi Hospital  University of Tsukuba Hospital  Kasugai Municipal Hospital  Jikei University Kashiwa Hospital  Nagoya Medical Center  National Defense Medical College Hospital  Sakai City Medical Center  Nara Prefecture General Medical Center  University Hospital Kyoto Prefectural University of Medicine  Fujisawa City Hosipital  Showa University Hospital  Sendai City Hospital  Tokyo Metropolitan Tama Medical Center  Mito Saiseikai General Hospital  Hiroshima University Hospital  Toyooka Public Hospital Tajima Emergency and Critical Care Medical Center  National Hospital Organization Kyoto Medical Center  Tohoku University Hospital  Japanese Red Cross Medical Center  Sakakibara Heart Institute  Naha City Hospital  Tsuchiura Kyodo General Hospital  Yokohama City Minato Red Cross Hospital  Tokyo Medical University Ibaraki Medical Center  Hyogo Emergency Medical Center  Fujita Health University  Takatsuki general hospital  Kanazawa University Hospital  Kagawa University  Yokosuka Kyosai Hospital  SUBARU Health Insurance Society Ota Memorial Hospital | Takako Akimoto  Moritoki Egi  Masataka Nakamura  Tetsuya Hara  Masahiro Takahashi  Nobuto Nakanishi  Fumiko Kambe  Yuta Arai  Hiroshi Kamijo  Takashi　Hongo  Masakazu Nitta  Kenzo Ishii  Yutaka Sakuda  Masahito Horiguchi  Munekatsu Miyahira  Tadashi Kaneko  Masaru Matsumoto  Ayaka Matsuoka  Hiromichi Naito, Hiroshi Morimatsu  Yuhei Irie  Tatsutoshi Shimatani  Naoki Tominaga  Kazuya Omura  Kyohei Miyamoto  Akiyoshi Nagatomi  Naoki Hayase  So Oishi  Shinya Yaguchi  Tetsuro Kamo  Takeshi Nishida  Junji Hatakeyama  Hajime Hayami  Kazushige Inoue  Mamoru Komatsu  Kazuma Watanabe  Satoshi Asai  Toshihiko Yokotani  Kensuke Nakamura  Yuka Mishima  Hiroyuki Suzuki  Yuki Enomoto  Toshimichi Takahashi  Tatsuhiko Abe  Yasunari Morita  Kohei Yamada  Junko Kimura  Tomoya Yamaguchi  Ayako Noguchi  Osamu Akasaka  Fumihito Kasai  Yoshinobu Kameyama  Jun Hamaguchi  Yoshiki Tamatsukuri  Kohei Ota  Daisuke Taniguchi  Satoru Beppu  Takuya Shiga  Fumiaki Ishikawa  Takumi Nagao  Daisetsu Yasumura  Kenji Oike  Kei Sugiki  Kunio Yanagita  Shinichi Ijuin  Tomoyuki Nakamura  Chihiro Takayama  Masaki Okajima  Tomonori Sugawara  Yutaka Usuda  Kazuki Akieda |
| KOREA, REP. | Inje University Busan Pail Hospital  Samsung Medical Center  Korea University Anam Hospital  Gyeongsang National University Changwon Hospital  Armed Forces Capital hospital  Eunpyeong St. Mary's Hospital  Wonkwang University Hospital | Sukyoon Lee  Chi Ryang Chung  Jae-Seung Jung  Ho Cheol Kim  Donghoon Kim  Sei Won Kim  Chul Park |
| LEBANON | Nini Hospital | Ibrahim Salah el Din |
| LIBYA | Tripoli Central Hospital  Aljalla hospital  Sabha medical center  El Khadra Hospital  Nephro center  Ibn Sina  Althowra hospital  Oncology hospital musrata  Military field isolation hospital / Ajdabiya  Tripoli Medical Hospital  Raiaina hospital  Martyr Attia alkaseh teaching hospital  Almogarif hospital  Alkhadra hospital  Benghazi Medical Center  Alshahid Attia Alkasah General Hospital  Tobruk medical center  Tripoli children hospital  Dar Alshifa Hospital  Alhadba alkhadra hospital  Brega General Hospital  Elmarj teaching hospital  Paediatric Benghazi Hospital  Elmarj teatching hospital  Zliten Medical Center  Misurata Central Hospital  Diabetes and endocrine hospital  Chest center  Airport road polyclinic  Nalut central hospital  Sorman Teaching Hospital  Mietiga hospital  Misurata Medical Center  Sebha Medical Center  Almwasfat Isolation Center | Eman Abdulwahed  Duha Milad Abdullah  Ibrahim Ellojli  Abdussalam mady  Khaled aboumreeqa  Ahmed Buimsaedah  Safa mohammed Alfadheel  Aiman Ali Salem  Elham Braieg  Safia Adam Mosa  Mohammed Abdelkabir  Sana Moussa Shagour  Salmin Ibrahim Matoug  Shoukrie I. Shoukrie  Rafiq Boozed  Almoatasemalzanaty  Wesam ebrahim  Aihab ben amoor  Hana M Al-Gataani  Sulayman Almabrouk Sulayman Meelad  Ghadah alarbish  Wejdan Ali Alhadi  Mohammed YAHYA  Fatimah majeed Ali  Aml Ahmed Egbeta  Abdaljalel-A-Alzwai  Hana alfaytouri Alwaer alkeelani  TAHA ABUBAKER  Adel Gessel  Mohammed Abdalraheem Huwaysh  Khalid M.G Mohammed  Surour Salem Almabrouk  Abdulmuez Abdulmalik  Malek Mohamed Abusannuga  Almajdoub Ali Mohammed Ali  Emad Amkhatirah  Mabroukah saeid alshamikh  Yousef ibrahim hamad  Salma Muftah Omran  Abobaker Elbarouni  Fatimah Mohammed Bin Alsagheer  Hasan Almusrati  Rema husien  Mona Masaud Amro  Abdulmueti Alhadi  Abdulkarim Aldoukali Babaa  Abdalmageed alsharif alghenai  Saedah Abdeewi  Abdurraouf abusalama |
| MALAYSIA | International Islamic University Malaysia Medical Centre  University of Malaya Medical Centre  Sarawak General Hospital (Paediatric ICU)  Sarawak General Hospital (Neurosurgery ICU)  Sarawak General Hospital (Adult General ICU-Covid Ward)  Sarawak General Hospital (Adult General ICU-Non Covid Ward)  Hospital Raja Permaisuri Bainun | Mohd Basri MAT-NOR  Nor'azim Mohd Yunos  Huong Nai Law  Richard Teo Soon Kiat  Wan Daud Wan Kadir  Be Kim Leong  Shivani Rajasegaran |
| MOROCCO | Hassan 2 hospital  Zouneir skirj  Haut Grand Atlas  Mohammed V Military Hospital Rabat  Military Hospital Moulay Ismail  Ibn sina rabat  Hôpital Al Farabi ( ex Hopital maurice losteau) | Ayoub Ait lahcen  Salma ait lachgar  A.e.assia elhachmi  Ait Bouachrine Sarah  Balkhi Hicham  Yousra Zouine  Khalifa omar  Mohammed Leknani |
| NAMBIA | Windhoek Central Hospital | Ndatiyaroo W. Agapitus |
| NEPAL | Nepal Mediciti Hospital | Rashmi Suvedi |
| NETHERLANDS | Gelre Hospitals  Franciscus Gasthuis en Vlietland | Marleen Flim  Victor van Bochove |
| NIGERIA | Abubakar Tafawa Balewa University Teaching Hospital | Musa Abubakar Madaki |
| OMEN | Khoula Hospital | John Massoud |
| PALESTINE | Al Shifa Hospital  Alia Govermental Hospital  Rafidiah Hospital | Muawia S.J. Alkhazendar  Ghassan Al-Saikaly  Mustafa Abu Jayyab  Sarah Amro  Othman Mustafa |
| PHILIPPINES | Asian Hospital and Medical Center | Joanne Robles |
| POLAND | University Clinical Center , Gdansk | Tomasz Zwolinski |
| PORTUGAL | Centro Hospitalar Vila Nova de Gaia/Espinho  Centro hospitalar universitário São João | Ana Rios  Ana Afonso |
| QATAR | Hamad General Hospital | Ahmed S. Humadi Alsheikhly |
| ROMANIA | Fundeni Clinical Institute | Dana R Tomescu |
| RUSSIA | FSBI «NATIONAL MEDICAL RESEARCH CENTER FOR OBSTETRICS, GYNECOLOGY AND PERINATOLOGY NAMED AFTER ACADEMICIAN V.I.KULAKOV» MINISTRY OF HEALTHCARE OF THE RUSSIAN FEDERATION | Alexey Pyregov |
| SAUDI ARABIA | Prince Sultan Miltary Medical City  Prince Sultan military hospital Taif region  KING SAUD HOSPITAL | Ghaleb A.Almekhlafi  Ibrahim fawzy ELgouhary Abdelfattah  Osama Sobh |
| SINGAPORE | Singapore General Hospital  Woodlands Health Campus  Khoo Teck Puat Hospital | Vimal Palanichamy  Jayachandran Balachandran  Tan Boon Chai Sunny |
| SOUTH AFRICA | Groote Schurr Hospital | Ranem Sherif |
| SPAIN | Vall Hebron Institute of Research  Hospital Universitario de Getafe  Hospital Clínic Universitari de València  Hospital Verge de la Cinta de Tortosa, Tarragona  Hospital Clínico Universitario Lozano Blesa (Zaragoza)  Hospital Francesc de Borja Gandia | No name available  Fernando Frutos-Vivar  Rafael Badenes  Ferran Roche-Campo  Herrero García, Sandra  Susana Isabel Gil Garcia |
| SUDAN | Al Hakiem hospital  Al-Mak Nimir University Hospital  khartoum Isolation Center  Yastabshiroon Hospital  Wad Medani Teaching Hospital | Amani Ibrahim Abakar Bargo  Ahmed Osama Ahmed Babikir  Nusaiba Hassan Mohamed Eltahir  Shahd Elsiddig Ali Suliman  Mahmoud Saleh |
| SWIZERLAND | Spital Bülach  Clinique Cecil,Hirslanden | Bernd Yuen  Fleisch Isabelle |
| SYRIA | Al-Mouwasat University Hospital  Sham hospital  Tishreen University Hospital  Islamic Hospital Amman | Mohammad Karam Chaaban  Ahmad Mahmoud Hmaideh  Alaa Hamdan  Mohammed Sultan Amaereh |
| TURKEY | Karadeniz Technical University and Trabzon Kanuni Hospital Zonuldak Atatürk State Hospital  Düzce University Hospital | Ahmet Eroglu  Mahmud Islam  Türkay Akbaş |
| UNITED ARAB EMIRATES | Prime Hospital | Dirar Abdallah |
| UNITED KINGDOM | Our Lady of Lourdes Hospital  Queen Elizabeth the Queen Mother Margate  Gateshead Foundation Trust  University Hospitals Coventry and Warwickshire NHS Trust  Blackpool Teaching Hospitals NHS Trust  Queen Elizabeth Hospital Birmingham  Medway Maritime Hospital | Tharwat Aisa  Tarek Metwally  Hatim Albirnawi  David McWilliams  Nicky Williams  Jonathan Weblin  Sarah Elliott |
| UNITED STATES | Keck Medical Center of USC  UC Davis Health  Comer Children's Hospital, University of Chicago  Interfaith Medical Center  University of California San Diego  Lincoln Medical Center  Vassar Brothers Medical Center | John Margetis  Sarina A. Fazio  Neelima Marupudi  Ramakanth Pata  Robert L. Owens  Mohammad Aldiabat  Mazin Shaikhoun, MD |
| VENEZUELA | Hospital Central "Dr. Miguel Pérez Carreño" | Ingrid T. von der Osten R |
| YEMEN | Zaid hospital Sana'a city | Rafat Ameen Mohammed Al-saban |

**e - Appendix 2. Hospital and ICU background questions**

**Survey of basic information of the hospital/ICU**

Dear Study Participants,

The aim of this study is to describe the implementation of daily ICU care, especially associated with the ABCDEF bundle, PADIS guidelines, and nutrition for all ICU patients regardless of COVID-19 infection status.

We appreciate your interest in participating in this online survey. Please look over the information below carefully before agreeing to participate by clicking **‘Agree to participate’** at the bottom.

1. This investigation consists of two surveys:

Survey of basic information of the hospital/ICU (17 questions, 3-5 minutes to complete)

Survey of evidence-based and supportive ICU care (21 questions, 3-5 minutes to complete per ICU patient).

1. **Do NOT forget** to save the **Facility Registration Number** you will be given when you complete this survey of basic information of the hospital/ICU. You will need to enter this number when you answer the survey for daily ICU care on 27 January 2021. If you forget the number, you need to complete this survey again to get a new Facility Registration Number. The Study Committee does not save and cannot give you the number to protect anonymity.
2. You will be given about 20 questions associated with the ICU care you are providing to patients on 27 January 2021 in the survey of daily ICU care. The questionnaire includes information about age, gender, and estimated Body Mass Index, which are collected as categorial variables. You need to complete one questionnaire about ICU care for each patient. It should take about 3 to 5 minutes per questionnaire.
3. Your participation in this survey is voluntary. If you decide to join the study and start to answer, you may withdraw at any point during the questionnaire for any reason before submitting your answers by pressing the ‘Submit’ button/closing the browser.
4. This project has been reviewed by, and received ethical approval from, the Saiseikai Utsunomiya Hospital Institutional Review Board in Japan [2020-69] as central institutions of this study. The ISIIC Study Committee is investigating ethical issues in collaboration with a lawyer in Japan. We believe that ethical review from each facility is not required according to ethical policies in Japan. This study is conducted via a survey that does not include personal information as defined in the Personal Information Protection Law in Japan and is therefore judged to meet the requirements for ethics review exemption in the ethical guidelines for medical research as described below.
5. The collected information does not include data that can be used to identify the facility or individual.
6. We do not use samples taken from the human body.
7. This is an observational study that does not involve any intervention or any burden on personnel.
8. Registered institutions definitely have the right to decide whether they choose to answer the questions.
9. The content of the questions will not cause psychological distress to the respondents. Respondents in registered facilities are guaranteed the right to refuse to answer the questionnaire and are not disadvantaged or coerced into answering the questionnaire if they do not return it.
10. This study is aiming to include participating sites mainly in Asia, Europe, and Africa. We may exclude data from the United States and Oceania because of regional issues. Even if your data is excluded from the primary analysis, the name of the representative will be included in the Acknowledgement.

However, please note that each institution outside of Japan must consider the necessity for ethical review in their institution.

**The name of one representative from each participating facility will be included as an acknowledgement in all study publications.**

If you have any questions or concerns about any aspect of this survey, please do not hesitate to contact to the ISIIC 2 study committee below. We will do our best to answer your query and resolve it.

**ISIIC 2 Study Committee Mail: isiic2@jsea2005.org**

The principal investigators of this study are: Keibun Liu M.D., Ph.D., Kensuke Nakamura M.D., Ph.D., Hajime Katsukawa PT, Ph.D., and Osamu Nishida, M.D., Ph.D.

If you agree to participate after reading the above, please click **“Agree to participate”** at the bottom of this web page to start the questionnaire. If you do not agree to participate in this survey, please click **“Disagree to participate”** at the bottom to close this web page.

- **Agree to participate**
- **Disagree to participate**

**For all questions, please select the one best answer unless otherwise indicated.**

**Responder identity**

1. What is your role in the ICU?

- Nurse (include nurse managers, directors, and critical care nurse specialists)
- Intensivist (Physician)
- Physician other than intensivist
- Dedicated Physiotherapist in the ICU
- Non-dedicated physiotherapist
- Respiratory therapist
- Other

**Hospital Characteristics**

1. Which country are you working in?
2. How many beds does your hospital have?

- n<200 beds
- 200≦ n＜400
- 400≦ n＜600
- 600≦ n＜800
- n≧800

1. Describe the academic affiliation of your hospital.

- University hospital
- University-Affiliated hospital
- Community hospital
- Others

**ICU Structure / Characteristics**

1. What is the type of ICU?

- Medical
- Medical-surgical (mixed)
- Surgical
- Cardiac surgical
- Neurologic
- Pediatric
- Others

1. Is your ICU managed as a tele-ICU by another hospital or ICU?

- Yes
- No

1. How many ICU beds does your ICU have?
2. How many ICU beds are **specifically designated for patients with COVID-19**?
3. What is the nurse-to-patient ratio in your ICU?

- 1
- 2
- 3
- 4
- ≧5

1. Are these professionals dedicated to your ICU? (Click all that apply)

- Nurses
- Intensivist
- Physiotherapist
- Occupational therapist
- Respiratory therapist
- Nutritionist / dietitian
- Pharmacist
- None

1. What is the number of visiting hours in your ICU for a family per day in following situations?

(1) ★ For a family before the COVID-19 pandemic. ★

- No visiting hours available
- 0< n <6 hours
- 6≦ n＜12
- 12≦n ＜18
- 18≦ n＜24
- No limitation on visiting hours

(2) ★ For a family of a patient WITHOUT COVID-19, after the COVID-19 ★pandemic started

- No visiting hours available
- 0< n <6 hours
- 6≦n ＜12
- 12≦n ＜18
- 18≦n ＜24
- No limitation on visiting hours

(3) ★　For a family of a patient WITH COVID-19 infection ★

- No visiting hours available
- 0< n<6 hours
- 6≦ n＜12
- 12≦ n＜18
- 18≦ n＜24
- No limitation on visiting hours

1. Who may enter the room of patients with COVID-19 infections under the infection control regulations of your hospital? (Click all that apply)

- Nurses (include nurse managers, directors, and critical care nurse specialists)
- Intensivists
- Physicians other than intensivists
- Dedicated Physiotherapist in the ICU
- Non-dedicated physiotherapists
- Occupational therapists
- Respiratory therapists
- Facility management, e.g. for cleaning
- No limitations / no regulations

1. How many times daily does your ICU have multi-professional/-disciplinary rounds to visit patients WITH COVID-19 infection?

- Not applicable
- At least once daily
- At least once a week
- once a month
- other

1. How many times daily does your ICU have multi-professional/-disciplinary rounds to visit patients WITHOUT COVID-19 infection?

- Not applicable
- At least once daily
- at least once a week
- once a month
- other

1. Are there written protocols shown below in your ICU? (Click all that apply)

- Pain management protocol (assess, prevent and manage Pain)
- Spontaneous Awakening Trial (SAT) management protocol
- Spontaneous breathing trial (SBT) management protocol
- Sedation management protocol
- Delirium management protocol (assess, prevent and manage Delirium)
- Early mobility and exercise protocol
- Family engagement and empowerment protocol
- Nutrition management protocol
- Physical restraint protocol
- ICU Diaries protocol
- No protocol
- Other

16. Who is primarily responsible for implementing the ABCDEF bundle in your ICU? If you have a primarily responsibility, it means that you take a key role to make decision on how the bundle will be provided to the patient.

- Multidisciplinary/-professional rounds / conference / team
- Nurse (include nurse managers, directors, and critical care nurse specialists)
- Intensivist (Physician)
- Physician (other than an intensivist)
- Physiotherapist (dedicated to the ICU)
- Physiotherapist (not dedicated to the ICU)
- Respiratory therapist
- No one has responsibility for implementing the bundle
- The ABCDEF bundle is not implemented in the ICU
- Others

17. What kinds of scale for ICU care do you use in your ICU? (Click all that apply)

- - Numerical Rating Scale (NRS)
  - Critical-care Pain Observation Tool (CPOT)
  - Behavioral Pain Scale (BPS)
  - Escala de Conductas Indicadoras de Dolor (ESCID)
  - Visual Analogue Scale for Pain assessment
  - Richmond Agitation- Sedation Scale (RASS)
  - Sedation-Agitation Scale (SAS)
  - Ramsay Sedation Scale
  - Confusion Assessment Method for ICU (CAM-ICU)
  - Intensive Care Delirium Screening Checklist (ICDSC)
  - Others
  - None

Caution！！

**Do NOT forget** to save the **Facility Registration Number** you will be given when you complete this survey of basic information of the hospital/ICU. You will need to enter this number when you answer the survey for daily ICU care on 27 January 2021.

If you forget the number, you need to complete this questionnaire again to receive another Facility Registration Number.

18. Is this questionnaire you just completed a repeat entry (for example if you lost the Facility Registry Number)?

- Yes
- No

**e - Appendix 3: Survey of evidence-based and supportive ICU care**

- You need to answer for all ICU patients.
- You need to complete one questionnaire (for ICU care) for each patient. For example, if you have three patients in your ICU, you need to complete three questionnaires.
- When you want to go to a questionnaire for the next patient, you must complete the current questionnaire first, then click “Next daily ICU care” or re-open the URL to answer for the next patient.
- Your colleagues can help by using the Facility Registration Number simultaneously to answer the survey about daily ICU care for other patients. For example, nurse A answers about ICU care for patient B, whereas doctor C answers about ICU care for patient D at the same time with the same Facility Registration Number.

For all questions, please select the one best answer unless otherwise indicated.

You do not need to answer these questions for patients receiving terminal or end of life care.

Facility Registration Number

1. Please write the Facility Registration Number you received after completing the hospital/ICU information questionnaire. If you forgot it, please complete the survey for basic information of hospital/ICU again and get a new facility registration number.

(survey of basic information is at URL: <https://forms.gle/aM7xdUHqZUiVNEDP8>)

Daily ICU care provided to this patient

1. Is this patient admitted to your ICU because of COVID-19 infection? COVID-19 is defined as a disease caused by a SARS-CoV-2 infection which is laboratory confirmed by RT-PCR.

Yes

No

1. How many days has this patient been in the ICU? (days)
2. What is the age of this patient (x)?

- x<20 years old
- 20≦x＜30
- 30≦x＜40
- 40≦x＜50
- 50≦x＜60
- 60≦x＜70
- 70≦x＜80
- x≧80

1. What is the patient’s gender?

- Male
- Female
- Other

1. What is the estimated Body Mass Index of your patient (x)?

- x<18.5
- 18.5≦x＜25
- 25≦x＜30
- 30≦x＜35
- x≧35

The questionnaire of daily ICU care starts here. Please answer this questionnaire based specifically on the daily ICU care you are providing or provided for this patient today, 27 January 2021.

1. What kind of respiratory assistance did the patient receive today? (click all that apply)

- No respiratory assistance
- Oxygen, such as nasal cannula, face mask, reserved face mask, and others
- Nasal high flow cannula
- Non-invasive ventilation
- Mechanical ventilation
- Veno-Venous Extracorporeal membrane oxygenation
- Veno-Arterial Extracorporeal membrane oxygenation
- Other

1. Choose the treatment you are giving to the patient today (click all that apply)

- Continuous/Intermittent renal replacement therapy
- Intra-Aortic Balloon Pumping (IABP)
- Impella^®^
- Continuous use of neuromuscular blockage
- Continuous use of vasoactive drugs
- Continuous use of analgesia agents
- Continuous use of sedation agents
- Other
- None of the above

1. What is the total number of hours of ‘prone positioning’ you provided for this patient today.

- Not applicable (e.g., because of no respiratory failure)
- 0 hours
- 0< x<6 hours
- 6≦x＜12
- 12≦x＜18
- 18≦x＜24
- 24 hours

1. What sedatives do you give continuously to this patient? (click all that apply)

- No sedative agents used
- Benzodiazepine
- Propofol
- Dexmedetomidine
- Barbiturate
- Inhaled sedation
- Remifentanil
- Other

Daily ICU care associated with the ABCDEF bundle and other supportive care

1. Did the patient receive following ICU care associated with the ABCDEF bundle today? (click all that apply)

- Regular standardized PAIN assessment using valid and reliable pain assessment scales (※１) 6 times or more per day.
- Spontaneous Awakening Trial (SAT) assessment (※２)
- Spontaneous Breathing Trial (SBT) assessment (※３)
  - Regular standardized SEDATION assessment using valid and reliable sedation assessment scales (※4) 6 times or more per a day.
- Regular standardized delirium assessment using valid and reliable delirium monitoring tools (※5) 2 times or more per day.
- Mobility activities that were out of bed or higher (※6) (It is equal to a score of 4 or higher according to the Intensive Care Unit Mobility Scale shown in Question 12)
- Family member/significant other of this patient is educated on the ABCDEF bundle and/or participate in at least one of the followings: rounds; conference; plan of care; or ABCDEF bundle related care, e.g., re-orientation, calming talks etc.
- The education and participation of family member/significant was conducted online.
- ICU diary (※7)
- Physical restraints on the bed at any time for the patient today

※１ The pain assessment scales include Numerical Rating Scale (NRS), Critical-care Pain Observation Tool (CPOT), Behavioral Pain Scale (BPS), and others.

※２ SAT is cessation of sedatives and narcotics or similar protocol to evaluate consciousness

※３ SBT is to turn the respiratory rate to zero with 8 or less of pressure support ventilation or similar local protocol to evaluate whether the patient meets the requirements for extubation

※４ The sedation assessment scales include Richmond Agitation- Sedation Scale (RASS), Sedation-Agitation Scale (SAS), Ramsay Sedation Scale, and others.

※５ The delirium assessment tools include Confusion Assessment Method for ICU (CAM-ICU), Intensive Care Delirium Screening Checklist (ICDSC), and others.

※６ i.e., dangling at edge of bed, standing at side of bed, walking to bedside chair, marching in place, walking in room or hall.

※7 An ICU diary is a patient journal, written by staff and families for several purposes, and includes daily entries about what happened.

1.
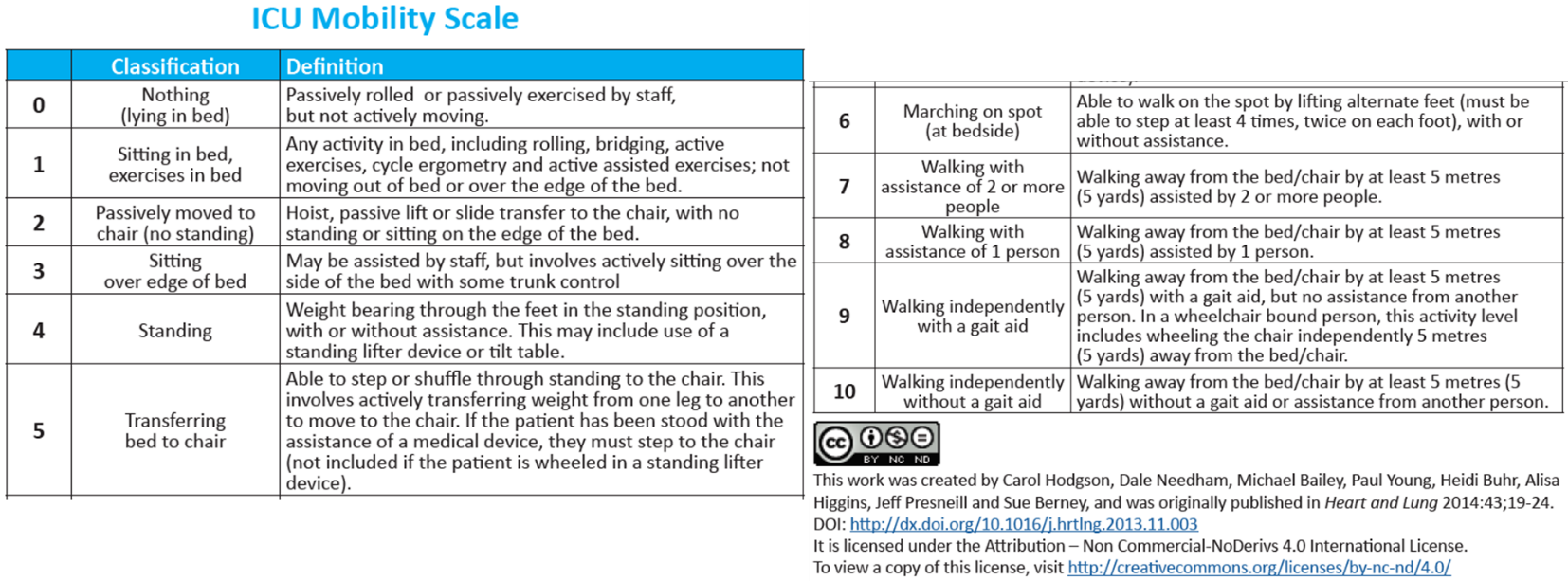
What was the highest mobility level of the patient today according to the Intensive Care Unit Mobility Scale? (See below- Intensive Care Unit Mobility Scale)
2. For days with limited mobility, select why she/he did NOT achieve the mobility level of sitting over edge of bed or more which is equal to a score of 4 or higher according to the Intensive Care Unit Mobility Scale. (Choose the most important barriers to rehabilitation)

- The Intensive Care Unit Mobility Scale was a score of 4 or higher
- Consciousness factor (existing consciousness disorder, RASS: ≤ -3 or ≥+2, deep sedation, delirium, etc.)
- Subjective symptoms (respiratory distress, BPS or > 3 or NRS > 5, fatigue, patient refusal, etc.)
- Respiratory factor (SpO2: <90%; FIO2: >0.6; respiratory rate: >30 times/min、ventilator unsynchronized, etc.)
- Circulatory factor (systolic blood pressure: <90 or >180 mmHg; mean blood pressure: <65 or >110 mmHg; heart rate: <50 or >120 beats/min; new arrhythmias; additional administration of vasopressors, etc.)
- Device factor (exist catheter, drain, dialysis, mechanical ventilation, or extracorporeal membrane oxygenation, etc.)
- Medical staff factor (lack of staff, holidays, many examinations, poor time adjustment, etc.)
- Factors associated with COVID-19 (restriction for medical staff to contact with the patients, restriction for rehabilitation, infectious control, etc.)
- Others

1. Do you have a target or goal on following today’s ICU care? (Click all that apply)

- Pain (i.e. targeted number on the pain scale)
- Sedation (i.e. targeted number on the sedation scale)
- Mobilization / Rehabilitation (i.e. targeted rehabilitation level)
- Nutrition (i.e. targeted energy or protein provision)
- Other
- No target or goal on ICU care

1. Was the patient diagnosed with delirium by the assessment tool, such as Confusion Assessment Method for ICU (CAM-ICU), Intensive Care Delirium Screening Checklist (ICDSC), and others? (put yes if positive on either assessment that day)

- No tool used for delirium assessment
- Yes
- No
- Not applicable (i.e. because of no consciousness)

1. Do you provide the patient with non-pharmacological interventions to control delirium today? (click all that apply)

- None
- Physical environment intervention (changing light application, earplugs, use of a mirror, acoustic or visual stimulation, restraint use avoidance)
- Orientation strategy
- Sedation reducing (spontaneous awaking trials, stop use of benzodiazepine or narcotics, etc)
- Family participation (orientation in family’s voice, nurse‐facilitated family participation in psychological care, )
- Exercise program (introduction of standardized rehabilitation protocol, strengthen mobilization/rehabilitation duration, frequency, or intensity, etc)
- Cerebral hemodynamic improving (doppler and oximetry monitoring, angioplasty to optimize cerebral blood flow, optimize mean arterial blood pressure or oxygenation, etc)
- Support for senses (hearing aids/glasses)
- Maximize sleep condition / standardized sleep protocol
- Sunbathing
- Multi-component program (implementation of ABCDEF bundle, combination of two or more interventions above)
- Other interventions

1. What mobility device/devices were used for this patient today? (click all that apply)

- No device
- Portable ergometer on the bed
- Neuro Muscular Electrical Stimulation
- Lift up device, ceiling lifter
- Tilt belt
- Walker
- Hip and Ankle Linked Orthosis (HALO)
- Others

1. Could the family meet with this patient today or see the patient using a monitor such as a phone and video today? (click all that apply)

- No
- In person
- Visiting through the glass outside the room
- Using an electronic device (using a monitor such as phone / video)

1. How do you provide nutrition for the patient? (click all that apply)

- Total parenteral nutrition
- Enteral nutrition (non-oral)
- Oral
- No nutrition

1. Total energy (kcal) of nutrition (x) provided within the last 24 hours (from yesterday until this morning) (In case of oral nutrition, please estimate based on the actual amount of intake)

- ＜10 (kcal/kg)
- 10≦x＜20 (kcal/kg)
- 20≦x＜30 (kcal/kg)
- x≧30 (kcal/kg)

1. Total protein (g/kg) provided within the last 24 hours (from yesterday until this morning) (In case of oral nutrition, please estimate based on the actual amount of intake)

- ＜1.2g/kg
- ≧1.2g/kg

We greatly appreciate your help and kindness.

You can complete the information of daily ICU care you provided to this patient by clicking “Submit” below. If you want to complete this survey for another patient with COVID-19, please click ‘Submit another response’ in the next page.

Submit

(Next page)

- If you want to complete this survey for another patient with COVID-19, please click ‘Submit another response’
- If you want to end the questionnaire or re-start later, please close this page.

**e - Appendix 4. Operational definitions of evidence-based and supportive ICU care**

| Elements of the ABCDEF bundle | Operational definition |
| --- | --- |
| Element A ^a,b^ | Regular standardized PAIN assessment using valid and reliable pain assessment scales six times or more per day. The pain assessment scales include Numerical Rating Scale (NRS), Critical-care Pain Observation Tool (CPOT), Behavioral Pain Scale (BPS), and others. |
| Element B ^a,b^ | Both SPONTANEOUS AWAKENING TRIALS and SPONTANEOUS BREATHING TRIALS. The spontaneous awakening trial is cessation of sedatives and narcotics or similar protocols to evaluate consciousness. The spontaneous breathing trial is to turn the respiratory rate to zero with eight or less of pressure support ventilation or similar local protocol to evaluate whether the patient meets the requirements for extubation. |
| Element C ^a,b^ | Regular standardized SEDATION assessment using valid and reliable sedation assessment scales six times or more per day. The sedation assessment scales include Richmond Agitation- Sedation Scale (RASS), Sedation-Agitation Scale (SAS), Ramsay Sedation Scale, and others. |
| Element D ^a,b^ | Regular standardized DELIRIUM assessment using valid and reliable delirium monitoring tools two times or more per day. The delirium assessment tools include Confusion Assessment Method for ICU (CAM-ICU), Intensive Care Delirium Screening Checklist (ICDSC), and others. |
| Element E ^a,b,c^ | MOBILITY activities that were out of bed or higher. It is equal to a score of 4 or higher according to the Intensive Care Unit Mobility Scale (i.e., dangling at edge of bed, standing at side of bed, walking to bedside chair, marching in place, walking in room or hall.). |
| Element F ^a,b^ | FAMILY ENGAGEMENT AND EMPOWERMENT that a family member/significant other of this patient is educated regarding the ABCDEF bundle and/or participates in at least one of the following: rounds; conference; plan of care; or ABCDEF bundle related care, e.g., re-orientation, calming talks etc. This element could be conducted in person or online. |

a. Pun BT, Balas MC, Barnes-Daly MA, Thompson JL, Aldrich JM, Barr J, et al. Caring for Critically Ill Patients with the ABCDEF Bundle: Results of the ICU Liberation Collaborative in Over 15,000 Adults. *Crit Care Med*. 2019;47:3–14.

b. Liu K, Nakamura K, Katsukawa H, Elhadi M, Nydahl P, Ely EW, et al. ABCDEF Bundle and Supportive ICU Practices for Patients With Coronavirus Disease 2019 Infection: An International Point Prevalence Study. *Crit Care Explor.* 2021;3:e0353.

c. Hodgson C, Needham D, Haines K, Bailey M, Ward A, Harrold M, et al. Feasibility and inter-rater reliability of the ICU Mobility Scale. *Heart Lung.* 2014;43:19–24.
